# Prediction of the potential geographical distribution of *Betula platyphylla* Suk. in China under climate change scenarios

**DOI:** 10.1371/journal.pone.0262540

**Published:** 2022-03-31

**Authors:** Wenliang Geng, Yanyan Li, Dongqi Sun, Bin Li, Pengyan Zhang, Hao Chang, Tianqi Rong, Ying Liu, Jingwen Shao, Zhenyue Liu, Huiru Zhu, Yuanyuan Lou, Qianqian Wang, Jinbing Zhang

**Affiliations:** 1 Key Laboratory of Geospatial Technology for Middle and Lower Yellow River Region, College of Environment and Planning, Research Center of Regional Development and Planning, Institute of Agriculture and Rural Sustainable Development, Henan University, Kaifeng, China; 2 Institute of Geographic Sciences and Natural Resources Research, Chinese Academy of Sciences, Beijng, China; Universiti Teknologi Malaysia, MALAYSIA

## Abstract

Climate is a dominant factor affecting the potential geographical distribution of species. Understanding the impact of climate change on the potential geographic distribution of species, which is of great significance to the exploitation, utilization, and protection of resources, as well as ecologically sustainable development. *Betula platyphylla* Suk. is one of the most widely distributed temperate deciduous tree species in East Asia and has important economic and ecological value. Based on 231 species distribution data points of *Betula platyphylla* Suk. in China and 37 bioclimatic, soil, and topography variables (with correlation coefficients < 0.75), the potential geographical distribution pattern of *Betula platyphylla* Suk. under Representative Concentration Pathway (RCP) climate change scenarios at present and in the 2050s and 2070s was predicted using the MaxEnt model. We analyzed the main environmental variables affecting the distribution and change of suitable areas and compared the scope and change of suitable areas under different climate scenarios. This study found: (1) At present, the main suitable area for *Betula platyphylla* Suk. extends from northeastern to southwestern China, with the periphery area showing fragmented distribution. (2) Annual precipitation, precipitation of the warmest quarter, mean temperature of the warmest quarter, annual mean temperature, and precipitation of the driest month are the dominant environmental variables that affect the potential geographical distribution of *Betula platyphylla* Suk. (3) The suitable area for *Betula platyphylla* Suk. is expected to expand under global warming scenarios. In recent years, due to the impact of diseases and insect infestation, and environmental damage, the natural *Betula platyphylla* Suk. forest in China has gradually narrowed. This study accurately predicted the potential geographical distribution of *Betula platyphylla* Suk. under current and future climate change scenarios, which can provide the scientific basis for the cultivation, management, and sustainable utilization of *Betula platyphylla* Suk. resources.

## 1. Introduction

Climate plays a major role in species distributions on regional and global scales, influencing the biodiversity and the potential geographical distribution of species [1–3]. Global warming has important impacts on community composition and structure, ecosystem function, biodiversity, and species distribution change [4–7]. Since the industrial revolution, built-up land has expanded [8], and land-use types have changed [9], and, under the joint action of human activities and natural factors, global warming has progressed [10]. According to the Fifth Assessment Report of the United Nations Intergovernmental Panel on Climate Change (IPCC), the global average temperature will increase by 0.3–4.8°C by the end of the 21st century (2081–2100) compared with that from 1986–2005 [11]. Under the background of global climate change, the geographical distribution range of species will also change. Thus, the prediction of the potential geographical distribution patterns of species under climate change scenarios has become a much-discussed issue in global change ecology and biogeography research [12, 13].

By studying the relationship between species distribution and environmental variables, we can explore the dominant environmental variables that affect the geographical distribution of species, determine the potential geographical distribution range of species, and analyze the impact of climate change on species distribution [14, 15]. Based on niche theory, the species distribution model is a powerful tool to evaluate the potential geographical distribution of species according to species distribution data and related environmental variables [16, 17]. At present, the commonly used models include the bioclimate analysis and prediction system (BIOCLIM), the genetic algorithm for rule-set prediction (GARP), random forests (RFs), and maximum entropy (MaxEnt) models [18–21]. Of these, the MaxEnt model is based on the theory of maximum entropy. A stable relationship between species and environment is determined by calculating state parameters with maximum entropy in the interaction system between species and environment to predict the potential geographical distribution of species [22, 23]. Compared with other models, the MaxEnt model is relatively simple and quick to run, with less sample requirement and stable operation. It can still perform well with either incomplete data or presence-only data [24, 25]. It has been widely used to simulate forest geographical distribution [26], assess flower habitat protection [27], evaluate the habitat suitability of wild protected animals [28], speculate species refuges [29], and simulate the distribution of suitable areas of medicinal materials [30].

*Betula platyphylla* Suk. is one of the most important pioneers and associated tree species in the Larix forest community in East Asia [31]. It is mainly distributed in Heilongjiang, Jilin, Liaoning, Inner Mongolia, Hebei, Henan, Shaanxi, Ningxia, Gansu, Qinghai, Sichuan, Yunnan, and Tibet, of China. *B*. *platyphylla* is distributed widely from northern to southern China and inhabits both pure and mixed forests. *B*. *platyphylla* has high medical value and commercial value, its buds are widely used in medicine, mainly as diuretics, sweating agents, analgesics [32, 33]. The latest research shows that the extraction of *B*. *platyphylla* buds may be a promising source of compounds with anti-cancer cytotoxic activity [34]. It has dense wood with a good white texture, which is widely used in furniture, building materials, and paper-making [35]. *B*. *platyphylla* is a kind of high-quality ecological forest. Scientific cultivation of *B*. *platyphylla* can not only increase vegetation coverage, reduce soil erosion, increase water storage, but also maintain the ecological balance of the forest. However, since the 1980s, due to the impact of pests and environmental damage, the natural birch forest in China has gradually narrowed. Thus, identifying the potential geographical distribution of *B*. *platyphylla* and predicting how climate change will affect its geographic range is necessary and meaningful.

Research on *B*. *platyphylla* has mainly focused on the influence of bioclimatic variables on the biomass of *B*. *platyphylla* forests [36], carbon storage of *B*. *platyphylla* forest ecosystems [37], and *B*. *platyphylla* multi-sanctuaries, multi-directional expansion, heterogeneous genetic models, etc. [29]. However, few scholars have predicted the potential geographical distribution pattern of *B*. *platyphylla* and its dominant environmental variables under future climate change scenarios. In this study, we collected and screened the species distribution data of *B*. *platyphylla*, based on soil, topography, and other related environmental data, using the MaxEnt model and ArcGIS 10.3 software spatial analysis function to simulate the current potential geographical distribution pattern of *B*. *platyphylla* according to the current climate data, explore its main environmental variables, and predict climate data for the 2050s and the 2070s. Then, we assessed the potential geographical distribution pattern of *B*. *platyphylla* in China in the future and its response to different climate change scenarios. This study provides a scientific basis for resource investigation and sustainable use of *B*. *platyphylla* and can serve as an important reference for future management and cultivation of *B*. *platyphylla* forests.

## 2. Materials and methods

### 2.1 Species occurrence data

**Fig 1:**
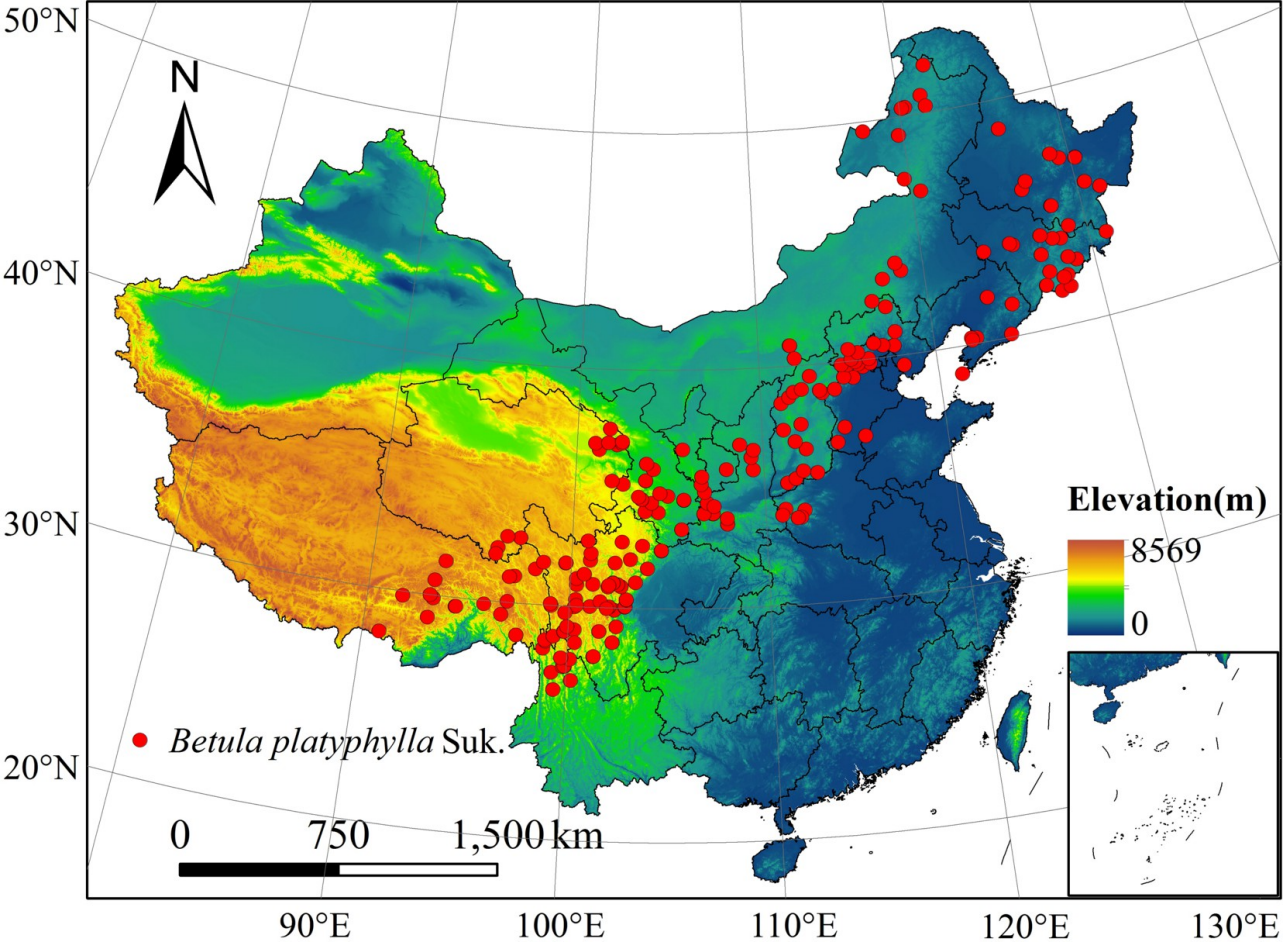
Distribution records of *Betula Platyphylla* Suk. in China. DEM was obtained from National Tibetan Plateau Data Center (http://data.tpdc.ac.cn). Reprinted from http://data.tpdc.ac.cn under a CC BY license, with permission from National Tibetan Plateau Data Center, original copyright [2019]. The boundary was obtained from Natural Earth (http://www.naturalearthdata.com/). Based on the principle of national and territorial integrity, we have modified and adjusted the vector boundary.

Through the Global Biodiversity Information Facility (https://www.gbif.org/), the National Specimen Information Infrastructure (http://www.nsii.org.cn/), and the Herbarium of the Institute of Botany, Chinese Academy of Sciences (http://pe.ibcas.ac.cn/), the species occurrence data of *B*. *platyphylla* from 1970 to 2020 were obtained (S1 Table). According to the following principles, reasonable species occurrence records were selected: Firstly, the occurrence records of *B*. *platyphylla* collected in this study did not include *Betula platyphylla* var. mandshurica or *Betula platyphylla* var. szechuanica, only *Betula platyphylla* Suk. Secondly, the species occurrence records must have complete longitude and latitude information to ensure geographical accuracy. For some sample occurrence records without geographical coordinates but have other detailed information, the Baidu coordinate picking system was used to obtain the corresponding latitude and longitude coordinates. Thirdly, some species occurrence data are sampled multiple times in different years, in which case only one record is kept. Fourthly, to match the environmental variables with a spatial resolution of 1 km × 1 km, the study area was divided into several 1km^2^ grids, and only one sample record was kept in each 1km^2^ grid [38]. These operations can greatly reduce the spatial autocorrelation of species occurrence data and effectively reduce the error. Finally, 231 occurrence records should be used for model operations (Fig 1).

### 2.2 Environmental variables

The 19 bioclimatic variables for the current and future scenarios were downloaded from the WorldClim dataset (http://www.worldclim.org/) [39]. The current climate dataset was generated by interpolation of observed weather data using a thin-plate smoothing spline during the period of 1970–2000 [21]. The Global climate model (GCM) data we use is based on the Coupled Model Intercomparison Project Phase 5 (CMIP5), compared with CMIP6 GCMs, CMIP5 GCMs still have a higher spatial resolution so far. The future climate scenarios were presented by 2050s (the average data for 2040–2060) data and 2070s data (the average data for 2060–2080) modeled by the Community Climate System Model version 4 (CCSM4) representing four future greenhouse gases concentration trajectories (RCP2.6, RCP4.5, RCP6.0, and RCP8.5). CCSM4 is one of the most effective GCMs for predicting the impact of future climate change on the distribution of animal and plant species and has been widely used in previous studies [40, 41]. RCP2.6, RCP4.5, RCP6.0, and RCP8.5 respectively represent low concentration, slightly lower concentration, slightly higher concentration, and high concentration greenhouse gas emission scenarios. Under this climate model scenario, by the end of this century (2081–2100), the global average temperature will increase by 0.3–1.7°C under RCP2.6 emission scenario, 1.1–2.6°C under RCP4.5 emission scenario, 1.4–3.1°C under RCP6.0 emission scenario, and 2.6–4.8°C under RCP8.5 emission scenario [11]. To avoid ignoring the subtle changes in species distribution caused by different climate change scenarios in the future, we chose to include RCP2.6, RCP4.5, RCP6.0, and RCP8.5 into the future potential geographic distribution of *B*. *platyphylla*. The data of the three topography variables are from National Tibetan Plateau Data Center (http://data.tpdc.ac.cn) digital elevation model of China. The data of 36 soil variables were obtained from the National Cryosphere Desert Data Center (http://www.ncdc.ac.cn). We used data from the world soil database established by the Food and Agriculture Organization and the International Institute for Applied Systems Analysis. The data source in China was 1:1,000,000 soil data from the Nanjing Soil Survey of the second national land survey [42]. The spatial resolution of the above environmental variables was 1 km. The vector boundary was obtained from Natural Earth (http://www.naturalearthdata.com/). Based on the principle of national and territorial integrity, we have modified and adjusted the vector boundary. Generally, most studies only select bioclimatic variables and topography variables for modeling, but soil variables are also important factors affecting species distribution. Stanton et al. (2012) have suggested better results could be achieved by combining important static variables with dynamic bioclimatic variables; this will produce better results than excluding static variables [43]. Therefore, in addition to bioclimatic variables and topography variables, we also added soil variables, and in our study, we assumed that soil and topography variables would not change during the simulation of potential geographic distribution in the context of climate change. For complete environmental variables, please refer to (S2 Table).

The multicollinearity of environment variables will affect the prediction results of the model, resulting in overfitting of model results [44]. Thus, correlation analysis and screening of environmental variables can improve model prediction accuracy. To eliminate the influence of multicollinearity on the model results, we take the following measures: Firstly, 58 environmental variables are tested by the Jackknife test in the MaxEnt model to evaluate the contribution rate of each variable, and the environmental variables with 0 contribution rate are eliminated. Secondly, the environmental variables with a contribution rate > 0 were selected, and the Spearman rank correlation test was conducted on soil environmental variables and climate environmental variables using SPSS ver. 21.0 (IBM Corp., Armonk, NY, USA). Environmental variables with a correlation coefficient < 0.75 were selected. For environmental variables with a correlation coefficient ≥ 0.75, only the environmental variables with the larger contribution rate were retained [45, 46]; those with a smaller contribution rate were excluded. Finally, 37 environmental variables were selected for the modeling analysis (Table 1).

**Table 1:** Percentage contribution rate and permutation importance value of the environmental variables used to predict the potential geographical distribution of *Betula platyphylla* Suk.

Data type	Variable	Percentage contribution rate (%)	Permutation importance value (%)
Bioclimatic variables	Bio1	7.00	4.47
	Bio2	1.27	3.14
	Bio3	1.54	1.41
	Bio5	2.12	1.97
	Bio10	7.85	3.77
	Bio12	4.66	18.00
	Bio13	0.14	10.32
	Bio14	5.82	9.71
	Bio15	1.07	1.10
	Bio16	0.03	0.20
	Bio18	33.71	8.26
	Bio19	0.34	1.03
Subtotal	—	65.54	63.39
Soil variables	AWC_CLASS	0.31	0.86
	DRAINAGE	0.50	0.05
	REF_DEPTH	0.68	0.68
	S_CASO_4_	0.02	0.00
	S_CEC_CLAY	2.62	0.72
	S_CEC_SOIL	0.41	0.20
	S_CLAY	0.37	1.59
	S_GRAVEL	1.46	1.79
	S_OC	0.04	0.02
	S_PH_H_2_O	1.08	3.16
	S_REF_BULK_DENSITY	0.64	0.38
	S_SAND	0.46	1.69
	T_BS	8.40	2.18
	T_CEC_CLAY	2.29	2.18
	T_CLAY	0.08	0.53
	T_ESP	0.12	0.76
	T_GRAVEL	0.90	1.68
	T_REF_BULK_DENSITY	0.02	0.00
	T_SILT	0.38	0.21
	T_TEB	0.59	1.17
	T_TEXTURE	0.32	0.36
	T_USDA_TEX_CLASS	0.32	0.62
Subtotal	—	22.01	20.83
topography variables	Elevation	4.50	12.05
	Slope	7.44	3.06
	Aspect	0.51	0.67
Subtotal	—	12.45	15.78
Total	—	100.00	100.00

### 2.3 MaxEnt modeling

In this study, the MaxEnt 3.4.1 (http://www.cs.princeton.edu/~schapire/maxent/) was selected for the simulation. The processed sample data of *B*. *platyphylla* distribution and 37 environmental variables after screening were imported into the MaxEnt model. Our modeling was performed according to the standard protocol for reporting species distribution models by Zurell et al (2020) [47].

The feature parameters were settled as Linear feature, Quadratic feature, Product feature, and Hinge feature, and “Create response curves”, “Make pictures of predictions” and “Do jackknife to measure variable importance” were chosen to interpret how individual variables affect the probability of the presence of *B*. *platyphylla*. In the basic part, the “Random test percentage” was set as 25, representing 75% of the sample data was randomly selected as the model training set; the remaining 25% of sample data was used as the test set to verify the model. The “Regularization multiplier” was set as 1 to prevent over-complexity and reduce overfitting by controlling the intensity of the chosen feature classes. The “Max number of background points” was set as 10000, the “Replicates” was set as 10. In the advanced part, the “Maximum iterations” was set as 500, the “Convergence threshold” was set as 0.0001. The output format was set as “Cloglog”, a previous study has shown that the “Cloglog” output was the optimal output mode for predicting the suitable area [48].

After the model was established, the area under the curve (AUC) of the receiver operating characteristic curve was used to evaluate the model accuracy [49–51]. AUC values ranged from 0 to 1, where larger AUC values represent better prediction results. The evaluation criteria were as follows: 0.50–0.60, prediction results fail, no credibility; 0.60–0.70, prediction results are poor and credibility is low; 0.70–0.80, prediction results are general, credibility is general; 0.80–0.90, prediction results are good and relatively reliable; 0.90–1.00, prediction results are very accurate and reliable. According to a previous study, models with AUC > 0.85 are sufficiently accurate to predict the potential geographical distribution of species under climate change scenarios [52].

### 2.4 Importance assessment of environmental variables

Among the output results of the MaxEnt model, the Jackknife method, percentage contribution rate, and permutation importance value can be used to evaluate the importance of environmental variables on the potential geographical distribution of *B*. *platyphylla*. The Jackknife method evaluates the importance of each environmental variable to the potential geographical distribution of species by comparing the differences among the output regularized training gain, regularized test gain, and AUC value [53]. According to the inherent algorithm, the coefficient corresponding to the eigenvalue is adjusted to improve the gain value of the model. The gain value is assigned to the environment variable depending on the eigenvalue and is converted into the contribution percentage, which is called the percentage contribution rate [54]. The permutation importance value is used to calculate the change range of the training AUC value through random permutation of the training set and normalize the result; the obtained percentage is the permutation importance value [55].

### 2.5 Division of suitable area and analysis of spatial pattern change

We used ArcGIS 10.3 software to divide and visualize the suitable area for *B*. *platyphylla*. Based on the maximum training sensitivity and specificity threshold (0.2932) generated from the MaxEnt model, the suitable area for *B*. *platyphylla* was classified [56, 57]: < 0.2932, unsuitable area; 0.2932–0.40, less suitable area; 0.40–0.60, moderately suitable area; and > 0.60, highly suitable area.

To more intuitively show the change in a suitable area for *B*. *platyphylla* combined with previous studies [58, 59], ArcGIS 10.3 software was used to convert the existing probability grid map of *B*. *platyphylla* into a binary map according to the threshold value (1 = suitable area, 0 = unsuitable area), overlay the distribution maps of different periods, and obtain the spatial change of *B*. *platyphylla* suitable area under the climate change scenarios using the grid calculator tool. In the output results, 0 represented a lack of present or future suitable area in a region, 1 represented a shrinkage of future suitable area, 2 represented the expansion of the future suitable area, and 3 represented stable present and future distribution of suitable area in a region.

## 3. Results and analysis

### 3.1 Accuracy evaluation of model prediction

The average AUC of testing data was 0.88, which is more than 0.85. The results showed that our model presented a high level of predictive performance and it can be used to predict the potential geographical distribution of *B*. *platyphylla* under climate change scenarios [60].

### 3.2 Main environmental variables affecting the potential geographical distribution of *B*. *platyphylla*

**Fig 2:**
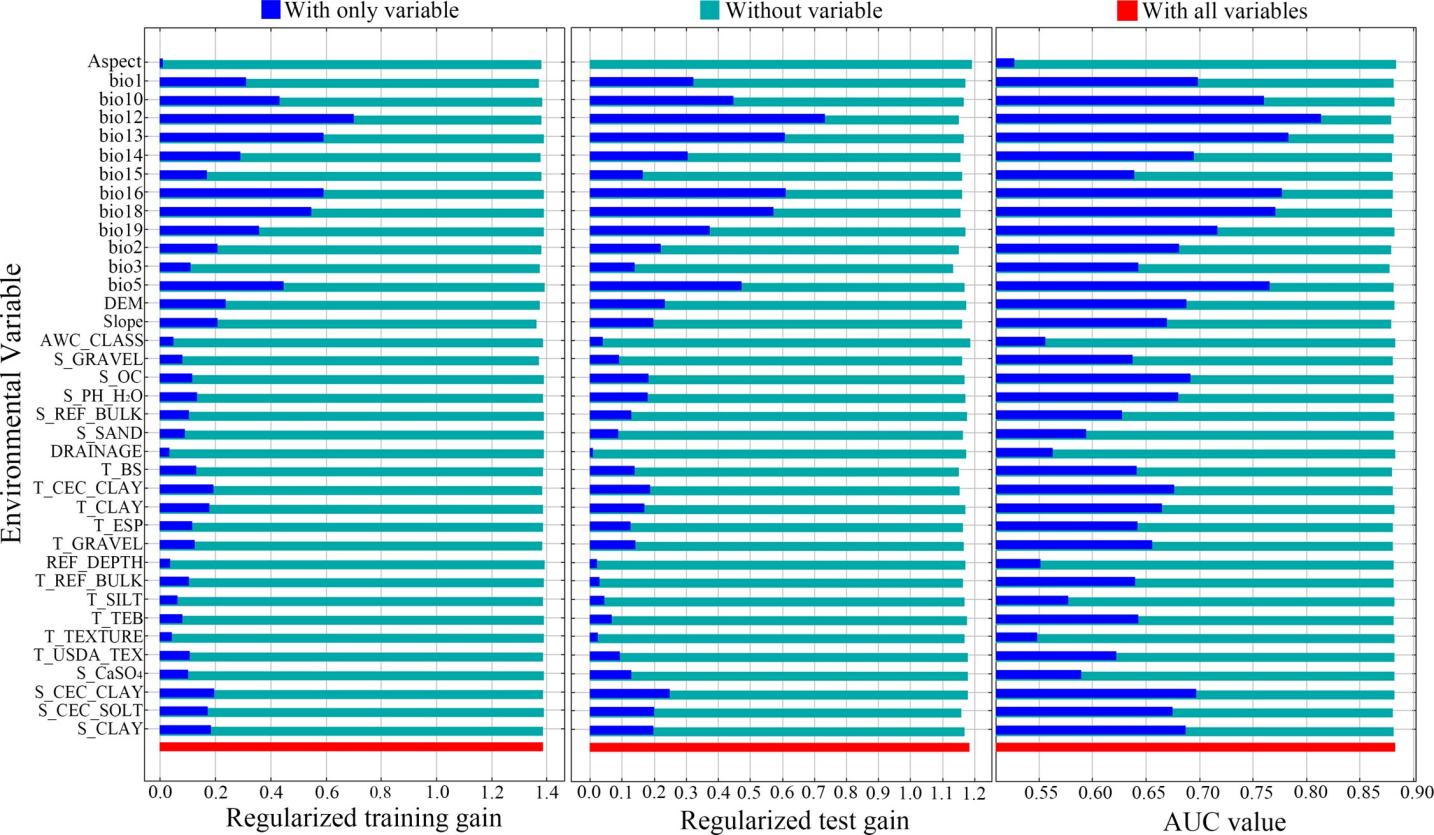
Results of the Jackknife test of environmental variables’ contribution in *Betula platyphylla* Suk.’s potential geographical distribution. The figure shows the result of the Jackknife test of variable importance using regularized training gain, regularized test gain, and AUC value on test data respectively. The blue bars indicate the gain using the solo environmental variable, the green bars indicate the gain excluding the single variable from the full model, and the red bars indicate the gain considering all variables.

Fig 2 presents the Jackknife test results. When only a single variable is used, the larger the regularization training gain, regularization test gain, AUC value, the more important the variable is to predict the potential geographical distribution of *B*. *platyphylla*. On the contrary, the closer the regularization training gain, regularization test gain, AUC value are to 0, the less important the variable is to predict the potential geographical distribution of *B*. *platyphylla*. When only a single environmental variable was applied, those with the largest regularized training gain were annual precipitation (Bio12; 0.70), precipitation of the wettest month (Bio13; 0.59), precipitation of the wettest quarter (Bio16; 0.59), precipitation of the warmest quarter (Bio18; 0.55), maximum temperature of the warmest month (Bio5; 0.45), mean temperature of the warmest quarter (Bio10; 0.43), precipitation of the coldest quarter (Bio19; 0.36), annual mean temperature (Bio1; 0.31), precipitation of the driest month (Bio14; 0.29), and elevation (0.24). When only single environmental variables were applied, those with the largest regularized test gains were Bio12 (0.73), Bio16 (0.61), Bio13 (0.61), Bio18 (0.57), Bio5 (0.47), Bio10 (0.45), Bio19 (0.37), Bio1 (0.32), Bio14 (0.30), and S_CEC_CLAY (0.25). When only single environmental variables were applied, the highest AUC values were obtained for Bio12 (0.81), Bio13 (0.78), Bio16 (0.78), Bio18 (0.77), Bio5 (0.77), Bio10 (0.76), Bio19 (0.72), Bio1 (0.70), S_CEC_CLAY (0.70), and Bio14 (0.70).

The percentage contribution rate and permutation importance value of environmental variables to MaxEnt modeling were obtained from the model output (Table 1). The percentage contribution rates of single environmental variables > 3% were Bio18 (33.71%), T_BS (8.40%), Bio10 (7.85%), slope (7.44%), Bio1 (7.00%), Bio14 (5.82%), Bio12 (4.66%), and elevation (4.50%); the cumulative contribution rate was 79.38%. The permutation importance values of single environmental variable > 3% were Bio12 (18.00%), elevation (12.05%), Bio13 (10.32%), Bio14 (9.71%), Bio18 (8.26%), Bio1 (4.47%), Bio10 (3.37%), S_PH_H_2_O (3.16%), Bio2 (3.14%), and slope (3.06%).

**Fig 3:**
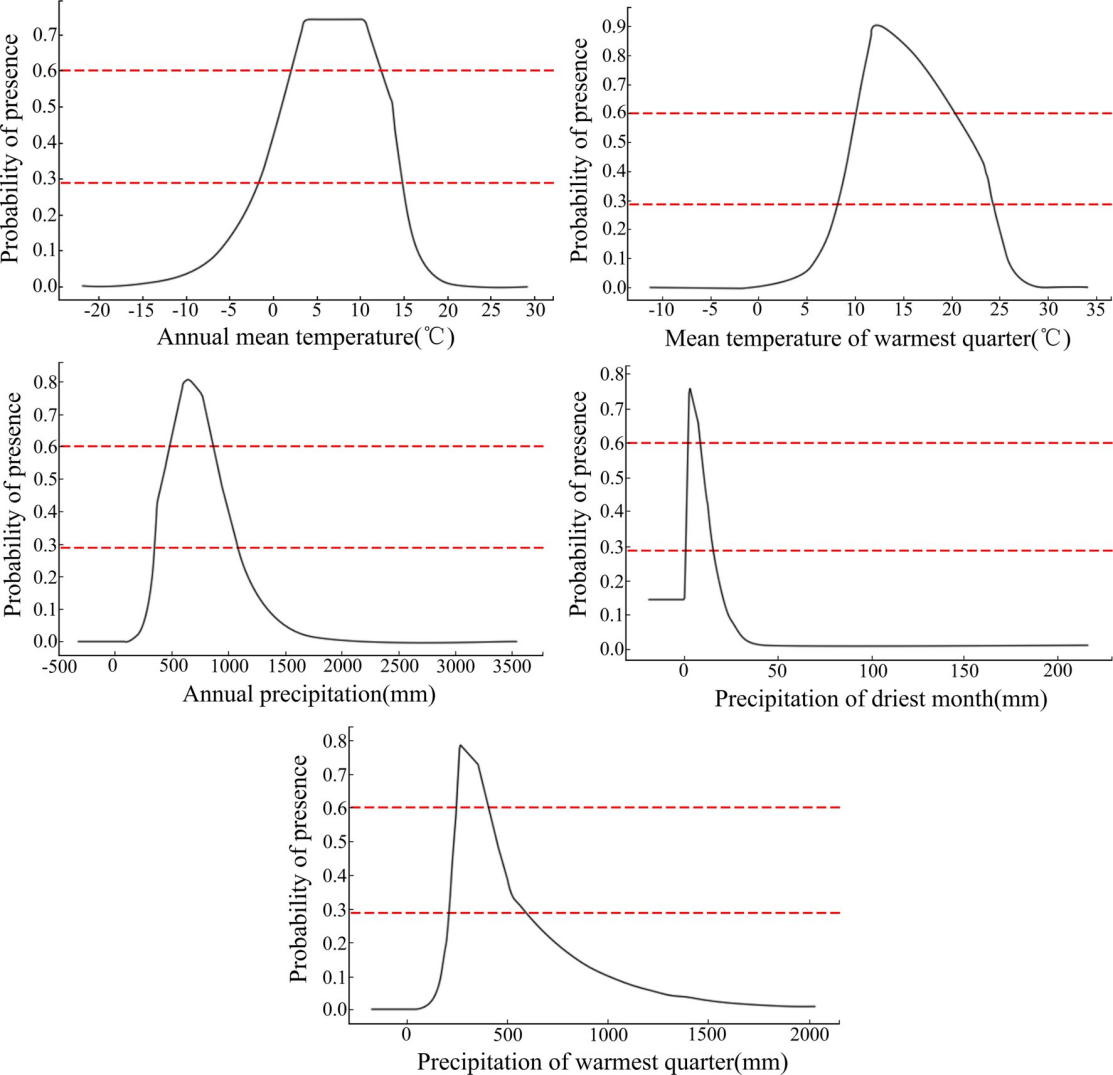
Response curve of the dominant environmental variables.

According to the Jackknife method, percentage contribution rate, and permutation importance values of the model output, Bio12, Bio18, Bio10, Bio1, and Bio14 were the main environmental variables affecting the potential geographical distribution of *B*. *platyphylla*. According to the response curve of environmental variables to the presence probability in the MaxEnt model (Fig 3), taking the presence probability greater than 0.2932 as the selection condition of suitable area for *B*. *platyphylla*, the threshold values of the dominant environmental variables affecting the distribution of suitable area for *B*. *platyphylla* were as follows: Bio12, 350–1075 mm; Bio18, 206.5–587 mm; Bio10, 8–24.3°C; Bio1, −2–14.7°C; and Bio14, 0–16 mm. Taking a presence probability greater than 0.6 as the selection condition, the threshold values of the dominant environmental variables affecting the distribution of highly suitable area for *B*. *platyphylla* were as follows: Bio12, 495–869 mm; Bio18, 239–369.6 mm; Bio10, 10–20.2°C; Bio1, 2–12.5°C; and Bio14, 1.4–8.2 mm.

### 3.3 Simulation of the potential geographic distribution of *B*. *platyphylla* under climate change scenarios

#### 3.3.1 Potential geographical distribution of *B*. *platyphylla* under current climatic conditions

**Fig 4:**
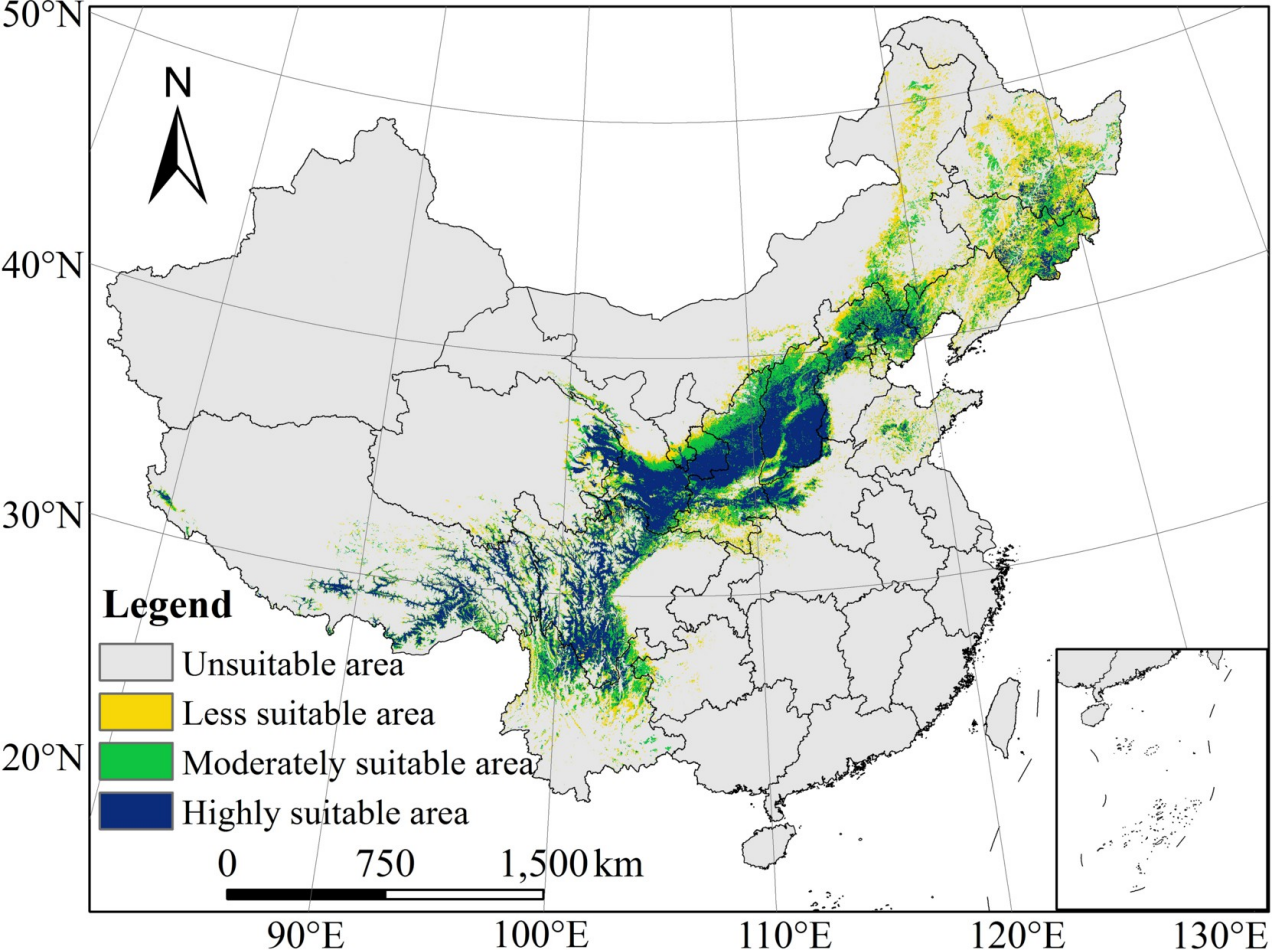
Distribution of suitable area for *Betula platyphylla* Suk. under current climate scenarios. (When presence probability is < 0.2932, unsuitable area; When presence probability is 0.2932–0.40, less suitable area; When presence probability is 0.40–0.60, moderately suitable area; And when presence probability is > 0.60, highly suitable area). The boundary was obtained from Natural Earth (http://www.naturalearthdata.com/). Based on the principle of national and territorial integrity, we have modified and adjusted the vector boundary.

We use the MaxEnt model to obtain the distribution of suitable area for *B*. *platyphylla*, and ArcGIS 10.3 software was used for classification to obtain suitable area for *B*. *platyphylla* under the current climate scenario (Fig 4). Suitable area for *B*. *platyphylla* was mainly distributed in the Changbai Mountains and Xiaoxing’an Mountains (including Heilongjiang Province, Jilin Province, and Liaoning Province) in Northeast China, the Greater Khingan Mountains, Yanshan Mountains, Taihang Mountains, and Lvliang Mountains (including Hebei Province, Shanxi Province, Beijing City, Tianjin city, and Inner Mongolia Autonomous Region) in North China, and the Qinling Mountains and Qilian Mountains (including Qinghai Province and Ningxia Hui Autonomous Region) in Northwest China. In addition, a small number of suitable area was found in Hubei, Guizhou, and Chongqing. The distribution of mountains in China is shown in the (S1 Fig).

We use ArcGIS 10.3 software to extract the area of different suitable areas (Table 2). The total suitable area for *B*. *platyphylla* was 168.75 × 10^4^ km^2^; less suitable area (52.05 × 10^4^ km^2^), moderately suitable area (55.28 × 10^4^ km^2^), and highly suitable area (61.42 × 10^4^ km^2^) accounted for 5.50%, 5.84%, and 6.49% of the total area, respectively. Among provinces, Sichuan Province had the largest total suitable area, accounting for 12.38% of the total suitable area in China, as well as the largest highly suitable area, accounting for 18.63% of the highly suitable area in China. Shaanxi Province had the largest moderately suitable area, accounting for 10.98% of the moderately suitable area. Heilongjiang Province had the largest less suitable area, accounting for 17.22% of the less suitable area. The simulation results of the present potential geographical distribution of *B*. *platyphylla* were generally consistent with the collected distribution points of *B*. *platyphylla*, it shows that the MaxEnt model is suitable for predicting the potential geographical distribution of *B*. *platyphylla*.

#### 3.3.2 Potential geographic distribution of *B*. *platyphylla* under future climate change scenarios

**Fig 5:**
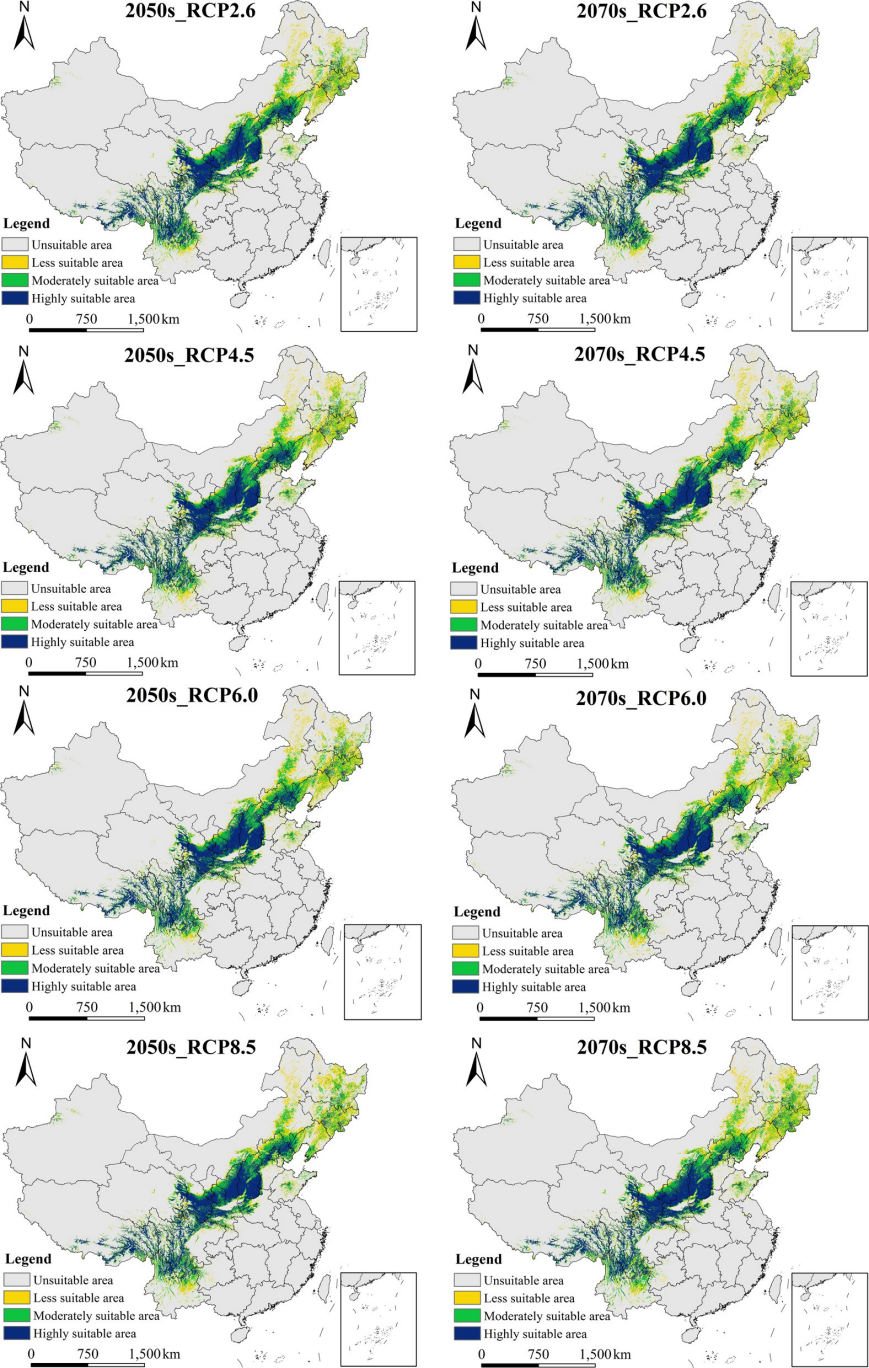
Distribution of suitable area for *Betula platyphylla* Suk. by Maxent Under the Four RCPs (RCP2.6, RCP 4.5, RCP 6.0, and RCP 8.5) in the Two Periods (the 2050s and 2070s). (When presence probability is < 0.2932, unsuitable area; When presence probability is 0.2932–0.40, less suitable area; When presence probability is 0.40–0.60, moderately suitable area; And when presence probability is > 0.60, highly suitable area). The boundary was obtained from Natural Earth (http://www.naturalearthdata.com/). Based on the principle of national and territorial integrity, we have modified and adjusted the vector boundary.

**Fig 6:**
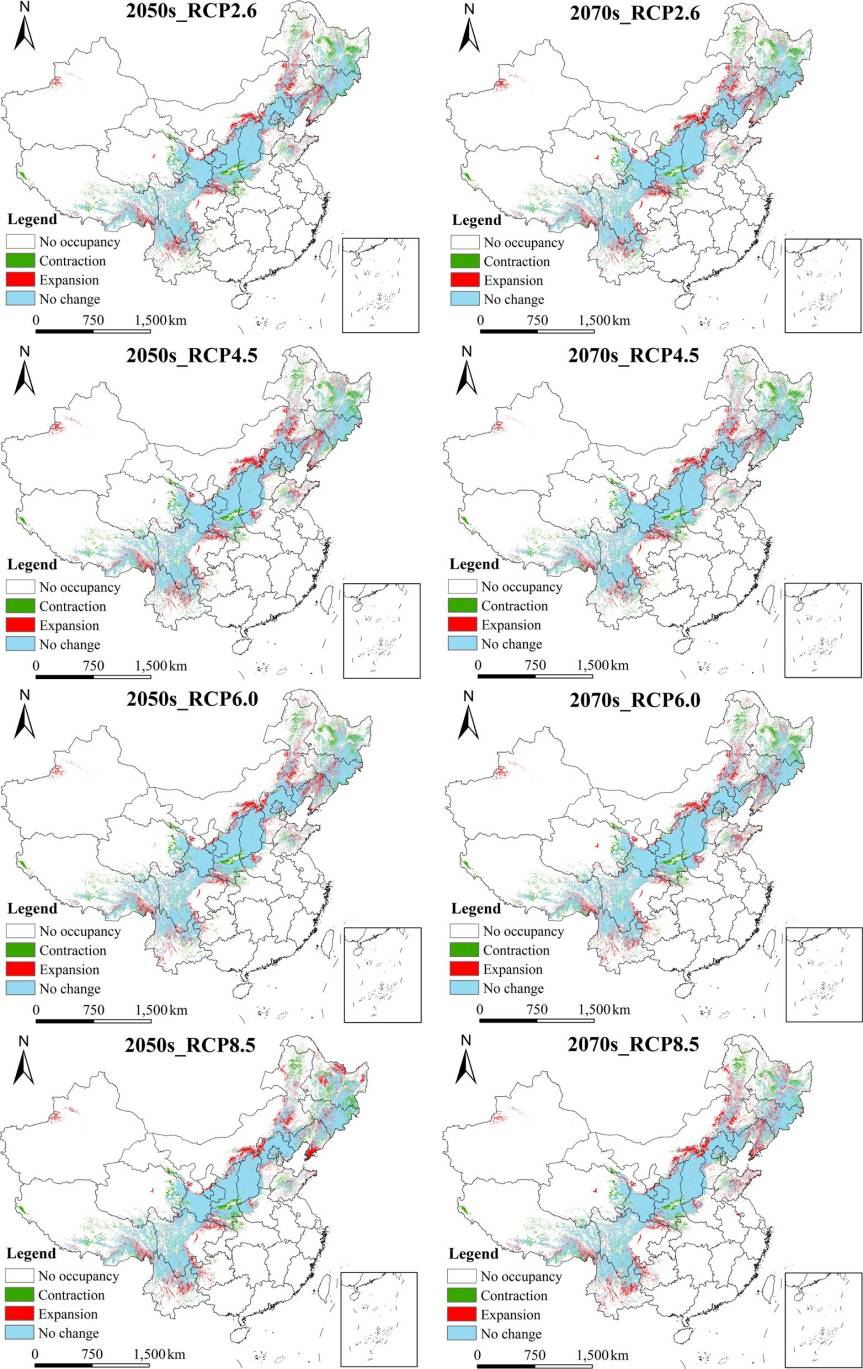
Changes of the suitable area of *Betula platyphylla* Suk. under the four RCPs (RCP2.6, RCP4.5, RCP6.0, and RCP8.5) in the Two Periods (the 2050s and 2070s), compared with the current potential geographic distribution. The boundary was obtained from Natural Earth (http://www.naturalearthdata.com/). Based on the principle of national and territorial integrity, we have modified and adjusted the vector boundary.

The classification tool of ArcGIS 10.3 software was used to grade the prediction results of the *B*. *platyphylla* suitability distribution model under future climate change scenarios, and maps of distribution (Fig 5) and changes in distribution (Fig 6) of suitable area for *B*. *platyphylla* under different climate scenarios in the 2050s and 2070s were obtained. Fig 5 shows the potential geographic distribution of *B*. *platyphylla* in the 2050s and 2070s under RCP2.6, RCP4.5, RCP6.0, and RCP8.5. The simulation results suggested similar distributions of suitable areas for *B*. *platyphylla* under the four RCP emission scenarios and for both the 2050s and 2070s, mainly in the Changbai Mountains, Greater Khingan Mountains, Yanshan Mountains, Taihang Mountains, Lvliang Mountains, Qinling Mountains, Qilian Mountains, Hengduan Mountains, hilly areas of central and southern Shandong Province, and Funiu Mountains. Under all scenarios, the suitable areas in Sichuan, Shaanxi, and Inner Mongolia accounted for more than 30% of the total suitable area. Table 2 also accurately shows the suitable area of *B*. *platyphylla* under future climate change scenarios, in the 2050s, under RCP6.0, the highly suitable area for *B*. *platyphylla* was 66.14 × 10^4^ km^2^; in the 2070s, RCP2.6 had the greatest range of highly suitable area for *B*. *platyphylla* (67.35 × 10^4^ km^2^) among the climate change scenarios.

**Table 2:** The proportion of suitable areas for *Betula Platyphylla* Suk. under different climate scenarios (%).

Period	RCPs	Unsuitable area	Less suitable area	Moderate suitable area	High suitable area
Current	——	82.18	5.50	5.84	6.49
2050s	RCP2.6	81.65	5.50	5.90	6.95
	RCP 4.5	81.26	5.68	6.09	6.97
	RCP 6.0	81.38	5.59	6.05	6.99
	RCP 8.5	81.25	5.77	6.05	6.93
2070s	RCP 2.6	81.41	5.56	5.92	7.11
	RCP 4.5	81.60	5.60	5.85	6.95
	RCP 6.0	81.36	5.65	5.98	7.01
	RCP 8.5	80.65	6.11	6.21	7.03

Under different climate change scenarios, the potential geographical distribution of *B*. *platyphylla* in the future was predicted to be relatively stable compared with the present distribution. Fig 6 and Table 3 presents the changes in the potential geographical distribution of *B*. *platyphylla* under different climate change scenarios between the present and the 2050s or 2070s. Under RCP2.6, RCP4.5, RCP6.0, and RCP8.5, from the present to the 2050s, the total suitable area for *B*. *platyphylla* increased by 2.95%, 5.16%, 4.49%, and 5.18%, respectively; and from the present to the 2070s, the total suitable area for *B*. *platyphylla* increased by 4.31%, 3.23%, 4.59%, and 8.56%, respectively. Under RCP2.6 and RCP8.5, the total suitable area in the 2070s was 2.27 × 10^4^ km^2^ and 5.70 × 10^4^ km^2^ higher than those in the 2050s, respectively. Under RCP4.5, the total suitable area in the 2070s was 3.24 × 10^4^ km^2^ lower than that in the 2050s. Under RCP6.0, the total suitable area in the 2070s changed little compared with that in the 2050s. In the future scenarios, the total suitable area for *B*. *platyphylla* tended to expand and was most evident in the southern section of the Greater Khingan Mountains, Yinshan Mountains, Changbai Mountains, Daba Mountains, Hengduan Mountains, and the eastern mountainous area of Qinghai Province. Shrinkage was most evident in the north of the Greater Khingan Mountains, the southeast of the Xiaoxing’an Mountains, the north of the Changbai Mountains, and the north of the Qinling Mountains. At present, there is no suitable area on the west side of the Tianshan Mountains, but there is a certain range of suitable areas in the 2050s and 2070s. On the west side of the Tianshan Mountains in Xinjiang, the total suitable area was largest under the 2070s RCP2.6 scenario and was 0.81 × 10^4^ km^2^, the total suitable area was the smallest area under the 2070s RCP4.5 scenario, and was 0.47 × 10^4^ km^2^.

**Table 3:** Changes of the suitable area of *Betula Platyphylla* Suk. under different climate scenarios (%).

Period	RCPs	Expansion	Contraction	No change	Total change
2050s	RCP2.6	15.12	12.16	87.84	2.96
	RCP 4.5	16.80	11.65	88.35	5.16
	RCP 6.0	15.60	11.12	88.88	4.49
	RCP 8.5	16.79	11.61	88.39	5.18
2070s	RCP 2.6	15.94	11.64	88.36	4.31
	RCP 4.5	15.13	11.90	88.10	3.23
	RCP 6.0	16.09	11.51	88.49	4.59
	RCP 8.5	18.72	10.16	89.84	8.56

## 4. Discussion

The output results of the MaxEnt model provide good reference values for important ecological issues, such as the prediction of species distribution and the impact of global warming on the suitable areas for species [61, 62]. Exploring the present and future potential geographical distribution of species is of great significance for the protection, use, and sustainable management of species in the context of global warming [63]. Based on the species distribution data of *B*. *platyphylla*, using the MaxEnt model, combined with different present climate change scenarios at present and in the 2050s and 2070s, this study predicted the potential geographical distribution of *B*. *platyphylla* under different climatic conditions and analyzed the dynamic changes in the suitable area. This will provide a reference for the cultivation and management of the *B*. *platyphylla* forest.

In any period of tree cultivation and growth, temperature and precipitation are always the most important driving factors. Temperature and water availability will affect the physiological activities and biochemical processes of trees. Combined with the prediction results of the model, under both present and future climate scenarios, the cumulative percentage contribution rates and cumulative permutation importance values of bioclimatic variables always exceeded 60%. Compared to soil and topographic variables, bioclimatic variables had the greatest impact on the potential geographical distribution of species. The cumulative contribution rates and cumulative replacement importance values of environmental variables related to precipitation were above 45%. The suitable area of *B*. *platyphylla* is mainly distributed in semi-humid and semi-arid areas. The vegetation distribution in this area has higher requirements for precipitation. However, due to the influence of latitude and altitude, the influence of temperature on the distribution of suitable areas is weakened. From another point of view, in the season of high temperature, precipitation can effectively alleviate the surface temperature. Thus, among bioclimatic variables, precipitation showed a great impact on the potential geographical distribution pattern of *B*. *platyphylla*. Based on the comprehensive analysis of the changes in the potentially suitable area and the most influential environmental variables of *B*. *platyphylla* in the 2050s and 2070s under four emissions scenarios, annual precipitation, precipitation of the warmest quarter, annual mean temperature, and mean temperature of the warmest quarter were positively correlated with a suitable area for *B*. *platyphylla*; increases in these variables were associated with an increase in the suitable area for *B*. *platyphylla*. Meanwhile, precipitation of the driest month was negatively correlated with the suitable area for *B*. *platyphylla*; a decrease in precipitation of the driest month was associated with an increase in the suitable area for *B*. *platyphylla*. For soil variables, topsoil base saturation、subsoil CEC (clay)、topsoil CEC (clay)、subsoil pH (H_2_O), and other variables have a certain impact on the suitable area of *B*. *platyphylla*, mainly because *B*. *platyphylla* is more suitable to grow in acidic soil, and soil moisture has different effects on *B*. *platyphylla* during different growth stages. If the soil moisture is too high, it will be harmful to *B*. *platyphylla* at the seedling stage, but it can effectively promote the growth of *B*. *platyphylla* at the growth stage. Different types of soil reflect different degrees of solar radiation, which affects the rate of photosynthesis and ultimately affects the growth of trees. According to the prediction results of the model, it can be seen that the altitude of 400–4500 m and the slope of 35° are suitable for the growth of *B*. *platyphylla*. Generally speaking, topography variable is an important driving factor of soil nutrients and water, so topography variables should be fully considered for the cultivation and planting of *B*. *platyphylla* forest.

Our research shows that the potential geographical distribution areas of *B*. *platyphylla* were mainly concentrated in the Changbai Mountains, Xiaoxing’an Mountains, Greater Khingan Mountains, Yanshan Mountains, Taihang Mountains, Lvliang Mountains, Qinling Mountains, Qilian Mountains, Hengduan Mountains, central and southern Shandong Mountains, and Funiu Mountains. This range is consistent with the results of Chen et al. [29]. The potential geographical distribution of *B*. *platyphylla* changed under different climate change scenarios. Relatively stable regions were mainly distributed in the Hengduan Mountains of western Sichuan, Shaanxi Province, Shanxi Province, southeastern Gansu Province, eastern Tibet, Jilin Province, Heilongjiang Province, and northern Hebei Province. Greater changes in suitable areas, typically expansions, were mainly observed in Greater Khingan Mountains, Xiaoxing’an Mountains, Changbai Mountains, Yinshan Mountains, Qinling Mountains, Daba Mountains, and Hengduan Mountains. Under the four climate change scenarios, the temperature increased to different degrees compared with the present, although the range of suitable areas for *B*. *platyphylla* remained similar. Under the current and future climate change scenarios, the prediction results of the potential geographical distribution of *B*. *platyphylla* were greater than the actual distribution. In the Changbai Mountains, Xiaoxing’an Mountains, and Greater Khingan Mountains forest areas in Northeast China, highly suitable areas accounted for 7.65% of the total highly suitable area in China. In this study, *Betula platyphylla* var. mandshurica was excluded from the research. Previous studies have found *B*. *platyphylla* has high genetic diversity, reflecting the genetic variation of *B*. *platyphylla* in Northeast China [64]. If the prediction is based on species major category, the results may be more consistent with the current actual distribution. However, this study does not intend to predict from the perspective of various species diversity, so only the sample data of *B*. *platyphylla* is considered. It can also fully reflect the niche represented by species distribution data is only a part of the actual ecosystem [65].

Many studies have shown that global warming will lead to the reduction or even total loss of suitable habitat for species [66–68]. In contrast, global climate change is predicted to increase the suitable area for *B*. *platyphylla*, consistent with the prediction of the potential geographical distribution of *Juglans regia* L. in China [69] and endangered medicinal plants in Yunnan [70]. This suggests that there will be more suitable areas for *B*. *platyphylla* cultivation in the future. *B*. *platyphylla* has a very high utilization value for humans, resulting in high market demand. Moreover, *B*. *platyphylla* forest is also of great significance to maintain the ecological balance of the forest. In the semi-arid area of the Loess Plateau, it can effectively improve the nutrient fixation capacity [71]. It is also very sensitive to salt stress, thus, breeding new varieties of *B*. *platyphylla* with high salt tolerance will help to improve the ecological environment in arid and saline-alkali areas [72]. *B*. *platyphylla* plays an important role in regional carbon sequestration. The annual net productivity and annual net carbon sequestration of *B*. *platyphylla* forest will increase with the increase of tree age [73]. Natural-based climate change emission reduction strategies have the potential to significantly reduce greenhouse gas emissions [74]. According to a report by the United Nations IPCC, development strategies such as afforestation, reforestation, and improved forest management can have key roles in the global emission reduction portfolio [75, 76]. Thus, the findings of this study could inform future governments and agencies about the most suitable area to cultivate birch forests to conserve water sources, beautify the environment, help alleviate global warming, and also bring higher economic benefits.

Compared with other studies, where analyses were limited to the effects of climate change on the potential geographical distribution of species in terms of bioclimatic variables [77–79], the novelty of this study lies in the comprehensive consideration of climate, soil, and topographic variables of the potential geographical distribution pattern of *B*. *platyphylla*. The soil physicochemical properties did not only include a few commonly used soil variables but relied on data from the world soil database. This study also has some limitations. The distribution of a species is not only impacted by climate, soil, and topography. Considering human activities related to land cover, hydrogeological conditions, road distribution, and residential distribution would also improve the accuracy of the simulation of the potential geographical distribution of *B*. *platyphylla*. The purpose of this study was to predict the potential geographical distribution of *B*. *platyphylla* in China on a large scale. According to the results of this study, small-scale field experiments were carried out in Northeast China, the Qinba Mountains, and the Hengduan Mountains, which are host to a wide distribution of suitable areas for *B*. *platyphylla*, to provide more accurate guidance for afforestation projects in China.

## 5. Conclusions

*B*. *platyphylla* is an important broad-leaved timber species in China with economic and ecological value. Based on species distribution data and environmental variables such as climate, soil, and topography, the current potential geographical distributions of *B*. *platyphylla* under different climate scenarios and that in the 2050s and 2070s were predicted. The main environmental variables influencing the geographical distribution were analyzed, and the range and change in suitable areas for *B*. *platyphylla* under different climate scenarios were compared. The results show that the suitable area of *B*. *platyphylla* in China extends from Xiaoxing’an Mountains in Northeast China to Hengduan Mountains in Southwest China. Under the climate warming scenario, the suitable area of *B*. *platyphylla* will further expand. Through artificial cultivation of *B*. *platyphylla* forest, we can optimize the structure of forestry development more reasonably and enrich the supply of forest resources. The function of carbon fixation and water conservation of *B*. *platyphylla* forest is of great significance for maintaining the ecological balance of forests. At the same time, the forest by-products will also produce considerable economic benefits. Our research will provide more accurate guidance for China to carry out afforestation projects, and also provide the scientific basis for investigation and sustainable utilization of *B*. *platyphylla* resources, and provide important references for management and cultivation of *B*. *platyphylla* forest.

## Supporting information

S1 Fig Distribution of mountains in China. DEM was obtained from National Tibetan Plateau Data Center (http://data.tpdc.ac.cn). Reprinted from http://data.tpdc.ac.cn under a CC BY license, with permission from National Tibetan Plateau Data Center, original copyright [2019]. The boundary was obtained from Natural Earth (http://www.naturalearthdata.com/). Based on the principle of national and territorial integrity, we have modified and adjusted the vector boundary.
(TIF)

S1 Table Dataset records of *Betula platyphylla* Suk. used for ecological modeling. (XLSX)

S2 Table Primitive environmental variables were used to predict the potential geographic distribution of *Betula platyphylline* Suk. (DOCX)

## References

[1] Mantyka-pringle CS, Martin TG, Rhodes JR. Interactions between climate and habitat loss effects on biodiversity: a systematic review and meta-analysis. Global Change Biology. 2012; 18(4): 1239–1252. 10.1111/j.1365-2486.2011.02593.x

[2] Burrows MT, Schoeman DS, Richardson AJ, Molinos JG, Hoffmann A, Buckley LB, et al. Geographical limits to species-range shifts are suggested by climate velocity. Nature. 2014; 507: 492–495. 10.1038/nature12976 24509712

[3] Wieczynski DJ, Boyle B, Buzzard V, Duran SM, Henderson AN, Hulshof CM, et al. Climate shapes and shifts functional biodiversity in forests worldwide. Proceedings of the National Academy of Sciences of the United States of America. 2019; 116: 587–592. 10.1073/pnas.1904390116 30584087 PMC6329988

[4] Alberto FJ, Aitken SN, Alia R, Gonzalez-Martinez SC, Hanninen H, Kremer A, et al. Potential for evolutionary responses to climate change evidence from tree populations. Global Change Biology. 2013; 19: 1645–1661. 10.1111/gcb.12181 23505261 PMC3664019

[5] Prăvălie R. Major perturbations in the Earth’s forest ecosystems. Possible implications for global warming. Earth-Science Reviews. 2018; 185: 544–571. 10.1016/j.earscirev.2018.06.010

[6] Thuiller W, Lavorel S, Araujo MB, Sykes MT, Prentice IC. Climate change threats to plant diversity in Europe. Proceedings of the National Academy of Sciences of the United States of America. 2005; 102: 8245–8250. 10.1073/pnas.0409902102 15919825 PMC1140480

[7] Chen W.Z.; Zhu D.; Ciais P.; Huang C.J.; Viovy N.; Kageyama M. Response of vegetation cover to CO_2_ and climate changes between Last Glacial Maximum and pre-industrial period in a dynamic global vegetation model. Quaternary Science Reviews. 2019; 218: 293–305. 10.1016/j.quascirev.2019.06.003

[8] Zhang PY, Yang D, Qin MZ, Jing WL. Spatial heterogeneity analysis and driving forces exploring of built-up land development intensity in Chinese prefecture-level cities and implications for future Urban Land intensive use. Land Use Policy. 2020; 99: 104958. 10.1016/j.landusepol.2020.104958

[9] Rong TQ, Zhang PY, Jing WL, Zhang Y, Li YY, Yang D, et al. Carbon Dioxide Emissions and Their Driving Forces of Land Use Change Based on Economic Contributive Coefficient (ECC) and Ecological Support Coefficient (ESC) in the Lower Yellow River Region (1995–2018). Energies. 2020; 13: 2600. 10.1016/j.quascirev.2019.06.003

[10] Ge QS, Wang F, Wang SW, Cheng BB. Certainty and uncertainty in global warming studies. Chinese Journal of Population, Resources and Environment. 2014; 24: 1–6.

[11] IPCC. Annex I: Atlas of Global and Regional Climate Projections. In Climate Change 2013: The Physical Science Basis. Contribution of Working Group I to the Fifth Assessment Report of the Intergovernmental Panel on Climate Change; Intergovernmental Panel on Climate Change: Cambridge, UK; New York, NY, USA, 2013; p. 1311.

[12] Bellard C, Bertelsmeier C, Paul L, Thuiller W, Courchamp F. Impacts of climate change on the future of biodiversity. Ecology Letters. 2012; 15(4): 365–377. 10.1111/j.1461-0248.2011.01736.x 22257223 PMC3880584

[13] Espíndola A, Pellissier L, Maiorano L, Hordijk W, Guisan A, Alvarez N. Predicting present and future intra-specific genetic structure through niche hindcasting across 24 millennia. Ecology Letters. 2012; 15(7): 649–657. 10.1111/j.1461-0248.2012.01779.x 22515791

[14] Shi H, Zhou Q, Xie FL, He NJ, He R, Zhang KR, et al. Disparity in elevational shifts of upper species limits in response to recent climate warming in the Qinling Mountains, North-central China. Science of The Total Environment. 2020; 706: 135718. 10.1016/j.scitotenv.2019.135718 31940727

[15] Pacifici M, Foden WB, Visconti P, Watson JEM, Butchart SHM, Kovacs KM, et al. Assessing species vulnerability to climate change. Nature Climate Change. 2015; 5: 215–224. 10.1038/nclimate2448

[16] Wiens JA, Stralberg D, Jongsomjit D, Howell CA, Snyder MA. Niches, models, and climate change: Assessing the assumptions and uncertainties. Proceedings of the National Academy of Sciences of the United States of America. 2009; 106: 19729–19736. 10.1073/pnas.0901639106 19822750 PMC2780938

[17] Austin M. Species distribution models and ecological theory: A critical assessment and some possible new approaches. Ecological modelling. 2007; 200(1–2): 1–19. 10.1016/j.ecolmodel.2006.07.005

[18] Busby JR. BIOCLIM-a bioclimate analysis and prediction system. Plant Protection Quarterly. 1991.

[19] Stockwell D. The GARP modelling system: Problems and solutions to automated spatial prediction. International Journal of Geographical Information Science. 1999; 13: 143–158. 10.1080/136588199241391

[20] Chambers D, Périé C, Casajus N, de Blois S. Challenges in modelling the abundance of 105 tree species in Eastern North America using climate, edaphic, and topographic variables. Forest Ecology and Management. 2013; 291: 20–29. 10.1016/j.foreco.2012.10.046

[21] Rong ZL, Zhao CY, Liu JJ, Gao YF, Zang F, Guo ZX, et al. Modeling the effect of climate change on the potential distribution of Qinghai Spruce (Picea crassifolia Kom.) in Qilian Mountains. Forests. 2019; 10(1): 62. 10.3390/f10010062

[22] West AM, Kumar S, Brown CS, Stohlgren TJ, Bromberg J. Field validation of an invasive species Maxent model. Ecological Informatics. 2016; 36: 126–134. 10.1016/j.ecoinf.2016.11.001

[23] Phillips SJ, Anderson RP, Schapire RE. Maximum entropy modeling of species geographic distributions. Ecological Modelling. 2006; 190(3–4): 231–259. 10.1016/j.ecolmodel.2005.03.026

[24] Zhang KL, Liu HN, Pan HL, Shi WH, Zhao Y, Li SL, et al. Shifts in potential geographical distribution of *Pterocarya stenoptera* under climate change scenarios in China. Ecology and Evolution. 2020; 10(11): 4828–4837. 10.1002/ece3.6236 32551064 PMC7297781

[25] Tognelli MF, Roig-Juñent SA, Marvaldi AE, Flores GE, Lobo JM. An evaluation for methods on modelling distribution of Patagonian insects. Revista Chilena de Historia Natural. 2009; 82: 347–360. 10.4067/s0716-078x2009000300003

[26] Chakraborty A, Joshi PK, Sachdeva K. Predicting distribution of major forest tree species to potential impacts of climate change in the central Himalayan region. Ecological Engineering. 2016; 97: 593–609. 10.1016/j.ecoleng.2016.10.006

[27] Zhang KL, Zhang Y, Zhou C, Meng JS, Sun J, Zhou TH, et al. Impact of climate factors on future distributions of Paeonia ostii across China estimated by MaxEnt. Ecological Informatics. 2019; 50: 62–67. 10.1016/j.ecoinf.2019.01.004

[28] Thapa A, Wu RD, Hu YB, Nie YG, Singh PB, Khatiwada JR, et al. Predicting the potential distribution of the endangered red panda across its entire range using MaxEnt modeling. Ecology and Evolution. 2018; 8(21): 10542–10554. 10.1002/ece3.4526 30464826 PMC6238126

[29] Chen TY, Lou AR. Phylogeography and paleodistribution models of a widespread birch (Betula platyphylla Suk.) across East Asia: Multiple refugia, multidirectional expansion, and heterogeneous genetic pattern. Ecology and Evolution. 2019; 9(13): 7792–7807. 10.1002/ece3.5365 31346441 PMC6635942

[30] Xu N, Meng FY, Zhou GF, Li YF, Wang B, Lu H. Assessing the suitable cultivation areas for *Scutellaria baicalensis* in China using the Maxent model and multiple linear regression. Biochemical Systematics and Ecology. 2020; 90: 104052. 10.1016/j.bse.2020.104052

[31] Lyu ZY, Yun RX, Wu T, Ma YJ, Chen ZJ, Jin YT, et al. Altitudinal differentiation in the radial growth of *Betula platyphylla* and its response to climate in cold temperate forest: A case of Oakley Mountain, Northeast China. Chinese Journal of Applied Ecology. 2020; 31(6): 1889–1897. doi: 10.13287/j.1001-9332.202006.011 34494741

[32] Shikov AN, Pozharitskaya ON, Makarov VG, Wagner H, Verpoorte R, Heinrich M. Medicinal plants of the Russian Pharmacopoeia; their history and applications. Journal of Ethnopharmacology. 2014; 154(3): 481–536. 10.1016/j.jep.2014.04.007 24742754

[33] Tolmacheva AA, Rogozhin EA, Deryabin DG. Antibacterial and quorum sensing regulatory activities of some traditional Eastern-European medicinal plants. Acta Pharmaceutica. 2014; 64: 173–186. 10.2478/acph-2014-0019 24914718

[34] Isidorov V, Szoka Ł, Nazaruk J. Cytotoxicity of white birch bud extracts: Perspectives for therapy of tumours. PLoS One. 2018, 13(8): e0201949. 10.1371/journal.pone.0201949 30106978 PMC6091957

[35] Liu Y, Xu HW, Shang FQ, Jiao H, Zhang LM, Luo JX, et al. Variation and Zoning of 16-Year-Old Betula platyphylla Provenance. Scientia Silvae Sinicae. 2016; 52(9): 48–56.

[36] Khan D, Muneer MA, Nisa Z-U, Shah S, Amir M, Saeed S, et al. Effect of climatic factors on stem biomass and carbon stock of Larix gmelinii and Betula platyphylla in Daxing’anling Mountain of Inner Mongolia, China. Advances in Meteorology. 2019; 2019: 1–10. 10.1155/2019/5692574

[37] Zheng T.; Mu C.C.; Zhang Y.; Li N.N. Effects of site condition on ecosystem carbon storage in a natural *Betula platyphylla* forest in the Zhangguangcai Mountains, China. Acta Ecologica Sinica. 2016; 36(19): 6284–6294.

[38] Xie GY, Olson DH, Blaustein AR. Projection of the global distribution of the emerging amphibian fungal pathogen, Batrachochytrium dendrobatidis, based on IPCC climate futures. PLoS One. 2016. 11(8): e0160746. 10.1371/journal.pone.0160746 27513565 PMC4981458

[39] Fick SE, Hijmans RJ. WorldClim 2: new 1-km spatial resolution climate surfaces for global land areas. International journal of climatology. 2017, 37, 4302–4315. 10.1002/joc.5086

[40] Bosso L, Luchi N, Maresi G, Cristinzio G, Smeraldo S, Russo D. Predicting current and future disease outbreaks of *Diplodia sapinea* shoot blight in Italy: species distribution models as a tool for forest management planning. Forest Ecology and Management. 2017, 400, 655–664. 10.1016/j.foreco.2017.06.044

[41] Abdelaal M, Fois M, Fenu G, Bacchetta G. Using MaxEnt modeling to predict the potential distribution of the endemic plant Rosa arabica Crép. in Egypt. Ecological informatics. 2019, 50, 68–75. 10.1016/j.ecoinf.2019.01.003

[42] Fischer G, Nachtergaele F, Prieler S, Velthuizen HT, Verelst L, Wiberg D. Global agro-ecological zones assessment for agriculture (GAEZ 2008) In: FAO Ia, editor. Laxemburg, Austria and Rome, Italy: 2008.

[43] Stanton JC, Pearson RG, Horning N, Ersts P, Reşit Akçakaya H. Combining static and dynamic variables in species distribution models under climate change. Methods in Ecology and Evolution. 2012, 3(2), 349–357. 10.1111/j.2041-210X.2011.00157.x

[44] Zhang KL, Yao LJ, Meng JS, Tao J. Maxent modeling for predicting the potential geographical distribution of two peony species under climate change. Science of the Total Environment. 2018; 634: 1326–1334. 10.1016/j.scitotenv.2018.04.112 29710632

[45] Moreno-Amat E, Mateo RG, Nieto-Lugilde D, Morueta-Holme N, Svenning JC, García-Amorena I. Impact of model complexity on cross-temporal transferability in Maxent species distribution models: An assessment using paleobotanical data. Ecological Modelling. 2015; 312: 308–317. 10.1016/j.ecolmodel.2015.05.035

[46] Zhang KL, Zhang Y, Tao J. Predicting the Potential Distribution of Paeonia veitchii (Paeoniaceae) in China by Incorporating Climate Change into a Maxent Model. Forests. 2019; 10(2): 190. 10.3390/f10020190.

[47] Zurell D, Franklin J, König C, Bouchet PJ, Dormann CF, Elith J, et al. A Standard Protocol for Reporting Species Distribution Models. Ecography. 2020; 43(5): 1–17. 10.1111/ecog.04960

[48] Phillips SJ, Anderson RP, Dudík M, Schapire RE, Blair ME. Opening the black box: An open-source release of Maxent. Ecography. 2017; 40(7): 887–893. 10.1111/ecog.03049

[49] Hanley JA, McNeil BJ. The meaning and use of the area under a receiver operating characteristic (ROC) curve. Radiology. 1982; 143(1): 29–36. 10.1148/radiology.143.1.7063747 7063747

[50] Fielding AH, Bell JF. A review of methods for the assessment of prediction errors in conservation presence/absence models. Environmental conservation. 1997; 24(1): 38–49. 10.1017/S0376892997000088

[51] Merow C, Smith MJ, Silander JA. A practical guide to MaxEnt for modeling species’ distributions: What it does, and why inputs and settings matter. Ecography. 2013; 36(10): 1058–1069. 10.1111/j.1600-0587.2013.07872.x

[52] Swets J. Measuring the accuracy of diagnostic systems. Science. 1988; 240(4857), 1285–1293. 10.1126/science.3287615 3287615

[53] Yang XQ, Kushwaha SP, Saran S, Xu J, Roy PS. MaxEnt modeling for predicting the potential distribution of medicinal plant *Justicia adhatoda* L. in Lesser Himalayan foothills. Ecological engineering. 2013, 51, 83–87. 10.1016/j.ecoleng.2012.12.004

[54] Zarzo-Arias A, Penteriani V, Delgado MDM, Torre PP, García-González R, Mateo-Sánchez MC, et al. Identifying potential areas of expansion for the endangered brown bear (Ursus arctos) population in the Cantabrian Mountains (NW Spain). PLoS One. 2019; 14(1): e0209972. 10.1371/journal.pone.0209972 30608946 PMC6319805

[55] Wei B, Wang RL, Hou K, Wang XY, Wu W. Predicting the current and future cultivation regions of *Carthamus tinctorius* L. using MaxEnt model under climate change in China. Global Ecology and Conservation. 2018; 16: e00477. 10.1016/j.gecco.2018.e00477

[56] Jiménez-Valverde A, Lobo JM. Threshold criteria for conversion of probability of species presence to either—or Presence—Absence. Acta Oecologica. 2007; 31(3): 361–369. 10.1016/j.actao.2007.02.001

[57] Weber TC. Maximum entropy modeling of mature hardwood forest distribution in four U.S. states. Forest Ecology and Management. 2011; 261(3): 779–788. 10.1016/j.foreco.2010.12.009

[58] Huang JH, Li GQ, Li J, Zhang XQ, Yan MJ, Du S. Projecting the range shifts in climatically suitable habitat for Chinese sea buckthorn under climate change scenarios. Forests. 2018; 9(1): 9. 10.3390/f9010009

[59] Zhang XQ, Li GQ, Du S. Simulating the potential distribution of *Elaeagnus angustifolia* L. based on climatic constraints in China. Ecological Engineering. 2018; 113: 27–34. 10.1016/j.ecoleng.2018.01.009

[60] Li YC, Li MY, Li C, Liu ZZ. Optimized Maxent Model Predictions of Climate Change Impacts on the Suitable Distribution of Cunninghamia lanceolata in China. Forests. 2020; 11(3): 302. 10.3390/f11030302

[61] Cao B, Bai CK, Zhang LL, Li GS, Mao MC. Modeling habitat distribution of *Cornus officinalis* with Maxent modeling and fuzzy logics in China. Journal of Plant Ecology. 2016; 9(6): 742–751. 10.1093/jpe/rtw009

[62] Zhang JJ, Jiang F, Li GY, Qin W, Li SQ, Gao HM, et al. Maxent modeling for predicting the spatial distribution of three raptors in the Sanjiangyuan National Park, China. Ecology and Evolution. 2019; 9(11): 6643–6654. 10.1002/ece3.5243 31236249 PMC6580265

[63] Porfirio LL, Harris RM, Lefroy EC, Hugh S, Gould SF, Lee G, et al. Improving the use of species distribution models in conservation planning and management under climate change. PLoS One. 2014; 9(11): e113749. 10.1371/journal.pone.0113749 25420020 PMC4242662

[64] Tsuda Y, Semerikov V, Sebastiani F, Vendramin G G, Lascoux M. Multispecies genetic structure and hybridization in the Betula genus across Eurasia. Molecular Ecology. 2017; 26(2): 589–605. 10.1111/mec.13885 27763698

[65] Pearson RG, Raxworthy CJ, Nakamura M, Peterson AT. ORIGINAL ARTICLE: Predicting species distributions from small numbers of occurrence records: A test case using cryptic geckos in Madagascar. Journal of Biogeography. 2007; 34(1): 102–117. 10.1111/j.1365-2699.2006.01594.x

[66] Thomas CD, Cameron A, Green RE, Bakkenes M, Beaumont LJ, Collingham YC, et al. Extinction risk from climate change. Nature; 2004: 427: 145–148. 10.1038/nature02121 14712274

[67] Ahmadi M, Hemami M-R, Kaboli M, Malekian M, Zimmermann NE. Extinction risks of a Mediterranean neo-endemism complex of mountain vipers triggered by climate change. Scientific reports. 2019; 9(1): 6332. 10.1038/s41598-019-42792-9 31004118 PMC6474857

[68] Wang WJ, Thompson FR Iii, He HS, Fraser JS, Dijak WD, Jones-Farrand T. Climate change and tree harvest interact to affect future tree species distribution changes. Journal of Ecology. 2019; 107(4): 1901–1917. 10.1111/1365-2745.13144

[69] Xu X, Zhang HY, Yue JJ, Xie T, Xu Y, Tian YL. Predicting Shifts in the Suitable Climatic Distribution of Walnut (Juglans regia L.) in China: Maximum Entropy Model Paves the Way to Forest Management. Forests. 2018; 9(3): 103. 10.3390/f9030103

[70] Yi YJ, Cheng X, Yang ZF, Zhang SH. Maxent modeling for predicting the potential distribution of endangered medicinal plant (H. riparia Lour) in Yunnan, China. Ecological Engineering. 2016; 92: 260–269. 10.1016/j.ecoleng.2016.04.010

[71] Jing GH, Hu TM, Liu J, Cheng JM, Li W. Biomass Estimation, Nutrient Accumulation, and Stoichiometric Characteristics of Dominant Tree Species in the Semi-Arid Region on the Loess Plateau of China. Sustainability. 2020; 12(1): 339. 10.3390/su12010339

[72] Xing BY, Gu CR, Zhang TX, Zhang QZ, Yu QB, Jiang J, et al. Functional Study of BpPP2C1 Revealed Its Role in Salt Stress in Betula platyphylla. Frontiers in plant Science. 2021; 11: 617635. 10.3389/fpls.2020.617635 33519877 PMC7841333

[73] Wei H, Man XL. Carbon storage and its allocation in *Betula platyphylla* forests of different ages in cold temperate zone of China. Chinese Journal of Plant Ecology. 2019, 43(10): 843–852.

[74] Adams DM, Alig RJ, McCarl BA, Callaway JM, Winnett SM. Minimum cost strategies for sequestering carbon in forests. Land economics. 1999; 75(3): 360–374. 10.2307/3147183

[75] Smith P, Bustamante M, Ahammad H, Clark H, Dong H, Elsiddig EA, et al. Agriculture, Forestry and Other Land Use (AFOLU). In: Edenhofer O, Pichs-Madruga R, Sokona Y, Farahani E, Kadner S, Seyboth K, et al., editors. Climate Change 2014: Mitigation of Climate Change: Contribution of Working Group III to the Fifth Assessment Report of the Intergovernmental Panel on Climate Change. Cambridge, United Kingdom, and New York City, USA: Cambridge University Press; 2014. p. 811–922

[76] Austin KG, Baker JS, Sohngen BL, Wade CM, Daigneault A, Ohrel SB, et al. The economic costs of planting, preserving, and managing the world’s forests to mitigate climate change. Nature Communications. 2020; 11: 5946. 10.1038/s41467-020-19578-z 33262324 PMC7708837

[77] Vieira KS, Montenegro PFG, Santana GG, Vieira WLdS. Effect of climate change on distribution of species of common horned frogs in South America. PLoS One. 2018;13(9): e0202813. 10.1371/journal.pone.0202813 30208067 PMC6135375

[78] Xu DP, Zhuo ZH, Wang RL, Ye M, Pu B. Modeling the distribution of *Zanthoxylum armatum* in China with MaxEnt modeling. Global Ecology and Conservation. 2019; 19: e00691. 10.1016/j.gecco.2019.e00691

[79] Wang R, Li Q, He S, Liu Y, Wang M, Jiang G. Modeling and mapping the current and future distribution of Pseudomonas syringae pv. actinidiae under climate change in China. PLoS One. 2018; 13(2): e0192153. 10.1371/journal.pone.0192153 29389964 PMC5794145

