# Supplementary figures and images for: Prediction of the potential geographical distribution of Betula platyphylla Suk. in China under climate change scenarios
Source: PLoS One. 2022 Mar 31;17(3):e0262540. doi: 10.1371/journal.pone.0262540 (PMC8970525; doi:10.1371/journal.pone.0262540)

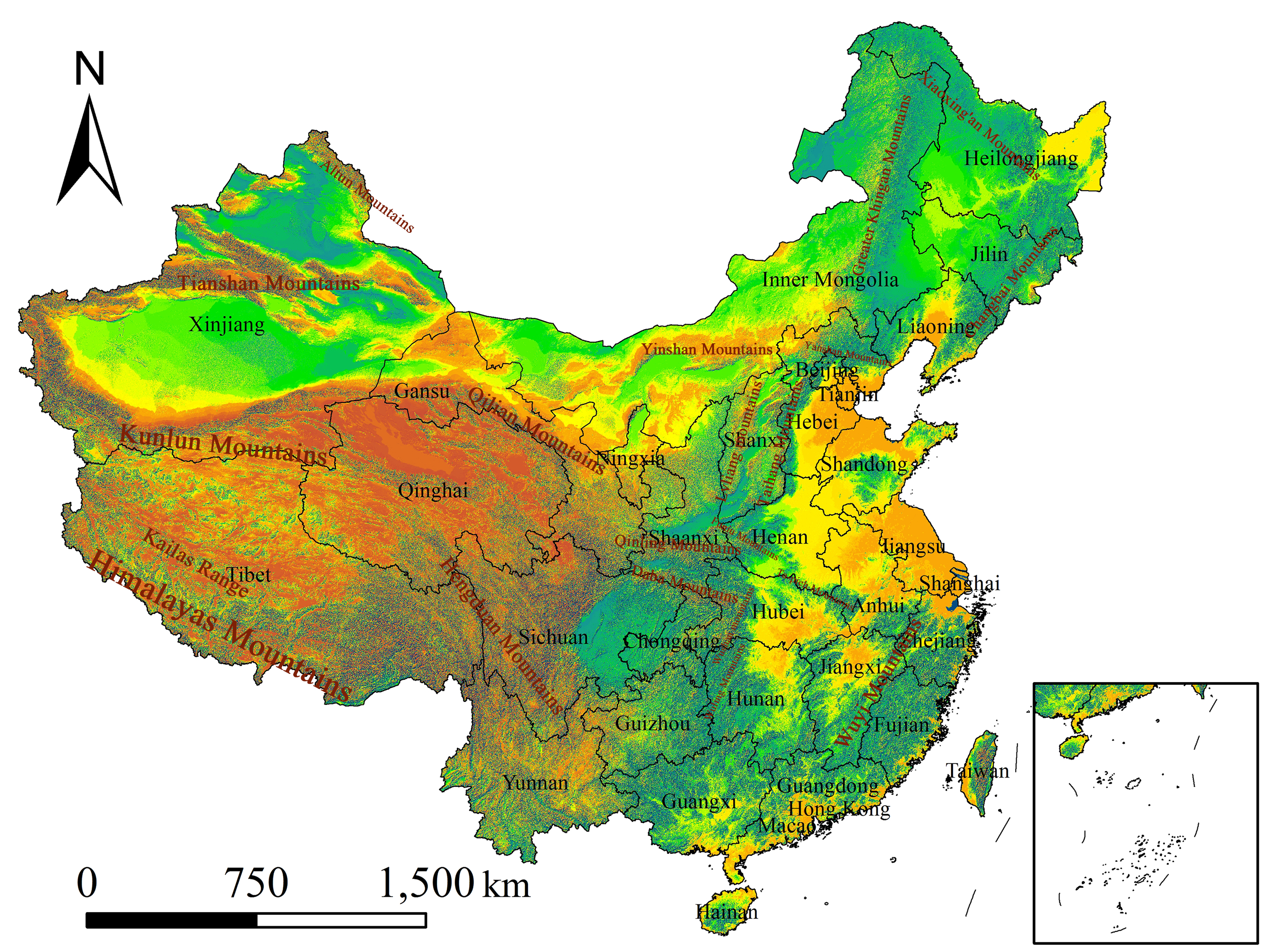

Supplement: S1 Fig — DEM was obtained from National Tibetan Plateau Data Center (http://data.tpdc.ac.cn). Reprinted from http://data.tpdc.ac.cn under a CC BY license, with permission from National Tibetan Plateau Data Center, original copyright [2019]. The boundary was obtained from Natural Earth (http://www.naturalearthdata.com/). Based on the principle of national and territorial integrity, we have modified and adjusted the vector boundary. (TIF) [file pone.0262540.s001.tif]
