# Supplementary material for: Prediction of the potential geographical distribution of Betula platyphylla Suk. in China under climate change scenarios
Source: PLoS One. 2022 Mar 31;17(3):e0262540. doi: 10.1371/journal.pone.0262540 (PMC8970525; doi:10.1371/journal.pone.0262540)
Supplement: S2 Table — (DOCX) [file pone.0262540.s003.docx]

**S2 Table. Primitive environmental variables used to predict potential geographic distribution of *Betula platyphylla* Suk.**

| **Data type** | **Variable** | **Description** | **Units** |
| --- | --- | --- | --- |
| Bioclimatic variables | Bio1 | Annual mean temperature | ℃ |
|  | Bio2 | Mean monthly temperature range | ℃ |
|  | Bio3 | Isothermality | ℃ |
|  | Bio4 | Temperature seasonality | ℃ |
|  | Bio5 | Max temperature of warmest month | ℃ |
|  | Bio6 | Min temperature of coldest month | ℃ |
|  | Bio7 | Temperature annual range | ℃ |
|  | Bio8 | Mean temperature of wettest quarter | ℃ |
|  | Bio9 | Mean temperature of driest quarter | ℃ |
|  | Bio10 | Mean temperature of warmest quarter | ℃ |
|  | Bio11 | Mean temperature of coldest quarter | ℃ |
|  | Bio12 | Annual precipitation | mm |
|  | Bio13 | Precipitation of wettest month | mm |
|  | Bio14 | Precipitation of driest month | mm |
|  | Bio15 | Precipitation seasonality | mm |
|  | Bio16 | Precipitation of wettest quarter | mm |
|  | Bio17 | Precipitation of driest quarter | mm |
|  | Bio18 | Precipitation of warmest quarter | mm |
|  | Bio19 | Precipitation of coldest quarter | mm |
| Soil variables | AWC_CLASS | AWC Range | code |
|  | DRAINAGE | Drainage class | code |
|  | REF_DEPTH | Reference Soil Depth | code |
|  | S_BS | Subsoil Base Saturation | % |
|  | S_CACO_3_ | Subsoil Calcium Carbonate | % |
|  | S_CASO_4_ | Subsoil Gypsum | % |
|  | S_CEC_CLAY | Subsoil CEC (clay) | cmol/kg |
|  | S_CEC_SOIL | Subsoil CEC (soil) | cmol/kg |
|  | S_CLAY | Subsoil Clay Fraction | % |
|  | S_ECE | Subsoil Salinity (ECe) | dS/m |
|  | S_ESP | Subsoil Sodicity (ESP) | % |
|  | S_GRAVEL | Subsoil Gravel Content | % |
|  | S_OC | Subsoil Organic Carbon | % |
|  | S_PH_H_2_O | Subsoil pH (H_2_O) | -log(H^+^) |
|  | S_REF_BULK_DENSITY | Subsoil Reference Bulk Density | kg/dm^3^ |
|  | S_SAND | Subsoil Sand Fraction | % |
|  | S_SILT | Subsoil Silt Fraction | % |
|  | S_TEB | Subsoil TEB | cmol/kg |
|  | S_USDA_TEX_ CLASS | Subsoil USDA Texture Classification | / |
|  | T_BS | Topsoil Base Saturation | % |
|  | T_CACO_3_ | Topsoil Calcium Carbonate | % |
|  | T_CASO_4_ | Topsoil Gypsum | % |
|  | T_CEC_CLAY | Topsoil CEC (clay) | cmol/kg |
|  | T_CEC_SOLT | Topsoil CEC (clay) | cmol/kg |
|  | T_CLAY | Topsoil Clay Fraction | % |
|  | T_ECE | Topsoil Salinity (Elco) | dS/m |
|  | T_ESP | Topsoil Sodicity (ESP) | % |
|  | T_GRAVEL | Topsoil Gravel Content | % |
|  | T_OC | Topsoil Organic Carbon | % |
|  | T_PH_H2O | Topsoil pH (H_2_O) | -log(H^+^) |
|  | T_REF_BULK_DENSITY | Topsoil Reference Bulk Density | kg/dm^3^ |
|  | T_SAND | Topsoil Sand Fraction | % |
|  | T_SILT | Topsoil Silt Fraction | % |
|  | T_TEB | Topsoil TEB | cmol/kg |
|  | T_TEXTURE | Topsoil Texture | / |
|  | T_USDA_TEX_CLASS | Topsoil USDA Texture Classification | / |
| Topography variables | Elevation |  | m |
|  | Slope |  | ° |
|  | Aspect |  | ° |
